# The Association between Obesity and Cognitive Function in Otherwise Healthy Premenopausal Arab Women

**DOI:** 10.1155/2018/1741962

**Published:** 2018-03-08

**Authors:** Abdulaziz Farooq, Ann-Marie Gibson, John J. Reilly, Nadia Gaoua

**Affiliations:** ^1^Athlete Health and Performance Research, Aspetar Orthopaedic and Sports Medicine Hospital, Doha, Qatar; ^2^School of Psychological Sciences and Health, University of Strathclyde, Glasgow, UK; ^3^School of Applied Sciences, London South Bank University, London, UK

## Abstract

**Objective:**

To examine the association between obesity and cognitive function in healthy premenopausal women.

**Methods:**

From a cohort of 220 women, 98 were randomly selected that provided complete data. Body composition was examined by dual-energy X-ray scan. All participants completed the Cambridge Neuropsychological Test Automated Battery (CANTAB) to assess cognitive performance in three domains: attention, memory, and planning executive function. The Reaction Time (RTI) test was used to assess motor and mental response speeds; the Stockings of Cambridge (SOC) test was used to assess planning executive function. For memory assessment, the Delayed Match to Sample (DMS), Pattern Recognition Memory (PRM), and Spatial Span (SSP) tests were used to assess forced choice recognition memory, visual pattern recognition memory, and working memory capacity, respectively.

**Results:**

36 (36.7%) were morbidly obese, 22 (22.4%) obese, and 23 (23.5%) overweight. Performance on RTI and SOC planning ability were not associated with body mass index (BMI). DMS mean time to correct response, when stimulus is visible or immediately hidden (0 ms delay), was higher by 785 ± 302 ms (milliseconds) (*p*=0.011) and 587 ± 259 ms (*p*=0.026) in morbidly obese women compared to normal weight women. Memory span length was significantly lower in overweight (5.5 ± 1.3, *p*=0.008) and obese women (5.6 ± 1.6, *p*=0.007) compared to normal weight (6.7 ± 0.9). DEXA-assessed body fat (%) showed similar associations as BMI, and latency to correct response on DMS and PRM was positively correlated with percentage of body fat, but not with VO_2_ max.

**Conclusion:**

In otherwise healthy premenopausal women, obesity did not impact accuracy on cognitive tasks related to attention, memory, or planning executive function, but morbid obesity was associated with higher latency to correct response on memory-specific tasks and lower memory span length.

## 1. Introduction

Cognitive function is a construct that represents an individual's ability to attain information and thus knowledge with constant application of memory, attention, and language skills [1]. While obesity is a well-known public health concern that leads to dyslipidemia, hypertension, type 2 diabetes, and cardiovascular diseases, there is limited research examining how it may influence cognitive function; however, several hypotheses have been put forward. These are pathophysiological changes with regard to vascular changes, insulin resistance, inflammation, reduced body fitness especially cardiovascular, and so on [2, 3]. Evidence suggests that the effect of obesity on cognitive function is not straightforward, rather obesity tends to affect the cognitive function of different people in different ways, specifically depending upon their age group. Lower body mass index (BMI) rather than higher BMI was found to be associated with increased incidence of dementia, especially in elderly people [4–8]. However, in midlife, the abovementioned findings were reversed; higher BMI was associated with increased incidence of dementia [9–11].

Some of the studies were observational, either longitudinal or cross-sectional studies, whereas some of them were designed to identify the underlying cellular processes [12–14]. Decrease in BMI in later years of life (postmenopausal women) is associated with poor cognitive performance. Driscoll et al. followed older postmenopausal women (65–79 years old) for an average of 3.5 years to examine the impact of weight gain on changes in cognition attributes. They found that women who maintained their weight or gained weight performed comparably on cognitive tests, but the women who lost weight during the study period had a poorer performance on cognitive tests, compared to either of the other two groups [15].

Conversely, studies have also concluded that there might not be any effect of obesity on cognitive function in females. For example, Elias et al. found that nonobese women and obese women scored comparably on various cognitive function tests (e.g., word fluency, visual reproduction, and digit span forward/backward), and the adverse outcome of obesity on cognitive function was limited to men only [16]. Some studies like that conducted by Kerwin et al. [17] were designed to explore the relationship between body fat distribution (measured by waist-to-hip ratio; WHR) and cognitive function. The researchers in this study classified older postmenopausal women by their BMI and found that the cognitive performance of women with a low waist-to-hip ratio decreased as their BMI increased, whereas the cognitive performance of women with a high waist-to-hip ratio increased as the BMI increased.

The majority of research is concentrated among menopausal women with underlying medical conditions. Therefore, further research is warranted especially in younger woman populations to explore the association between obesity and cognitive function by including more comprehensive and objective assessment of cognitive function as well as obesity. Again, BMI alone is an incomplete parameter to assess fatness; rather it should be supported by other parameters like fat distribution (assessed by dual-energy X-ray absorptiometry (DEXA) and computed tomography (CT) imagery) and cardiorespiratory fitness.

The aim of this study is to investigate the association between obesity and cognitive function in premenopausal healthy women.

## 2. Subjects and Methods

### 2.1. Subjects

We randomly recruited 220 women either from a gym (Aspire Active, Doha, Qatar) or by voluntary participation between February 2009 and December 2009. The study was conducted at Aspetar Qatar Orthopaedic and Sports Medicine Hospital. It was approved by the Institutional Research Ethics Committee, and all participants provided written consent prior to participation. The participants with diabetes mellitus, those who were pregnant or postmenopausal, or those receiving medical treatment for any chronic disease were excluded from the study; the only inclusion criterion was any willing healthy premenopausal women aged 18–50 years. The participants were clinically screened and excluded for the presence of diabetes mellitus, hypertension, or pregnancy. After 10-hour overnight fasting, the participants underwent a detailed clinical assessment, including body composition, fat distribution, and anthropometry measurements.

### 2.2. Anthropomorphic Assessment

Each participant's height was measured (without shoes) to the nearest 0.1 cm; weight was measured to the nearest 0.1 kg; and waist circumference was measured to the nearest 0.1 cm. A digital height-measuring device (Seca 242, Hamburg, Germany) and a portable stadiometer (Detecto, Webb City, Missouri, USA) were used to measure height and weight, respectively. Waist circumference was measured by a tape measurer horizontally at the smallest girth around the trunk. Two blood pressure readings were obtained 5 minutes apart with women in a relaxed seated position. The average systolic and diastolic blood pressures were calculated, and these values were used in subsequent analyses.

### 2.3. Body Composition Assessment

Fat mass (g), tissue (g), lean mass (g), and percentage of body fat were quantified by a DEXAD scanner XA (GE Medical Systems Lunar, Madison, Wisconsin, USA) uploaded with enCORE software (version 12.10; GE Medical Systems Lunar). CT scans were performed to obtain 5 CT axial images of each of the following regions: heart, liver, abdomen (at the L4-L5 level), and left and right midthighs. In the right thigh image, manual drawing was used to distinguish intramuscular adipose tissue from subcutaneous adipose tissue. For the abdominal region, cross-sectional axial images of the L4-L5 vertebral disc space were obtained. Manual drawing was used to differentiate omental adipose tissue from subcutaneous adipose tissue (which was further classified by manual drawing as superficial or deep). Two cross-sectional axial images of the left and right thighs at the femoral midpoint region were obtained. Intramuscular adipose tissue and subcutaneous adipose tissue in the thigh were distinguished by manual drawing using the right thigh image of adipose tissue. An upper limit of −30 Hounsfield units (HU) and a lower limit of −90 HU were used to differentiate adipose tissue from other tissue types on the CT images. All volumetric analyses were performed by an experienced radiologist by using the Somaris/5 Syngo CT2006A system (Siemens, Munich, Germany). The participants wore standard hospital gowns during the DEXA and CT scan procedures.

### 2.4. Fitness and Strength Assessment

The aerobic fitness of each participant was assessed by using the Bruce treadmill test. The starting speed was set to 2.74 km/h. The r and the intensity (speed and inclination) were increased by 2% at an interval of 3 minutes. The treadmill test was stopped once women requested to stop because of exhaustion. The peak heart rate (HR), percentage of predicted maximal HR, test duration, and peak oxygen uptake (i.e., VO_2_ max) were measured. Subjects were also evaluated for hand grip strength using a dynamometer (Lode BV, Groningen, Netherlands) and the Biodex 3.0 system, version 3.4 (Biodex Medical Systems, Inc., Shirley, NY, USA). Leg strength was assessed by the same Biodex system used to measure hand grip strength, in terms of isokinetic knee flexion and extension concentrically (at 30°/second and 120°/second, resp.) and isometric extension at 90° on the dominant leg.

### 2.5. Cognitive Assessment

The Cambridge Neuropsychological Test Automated Battery (CANTAB; Cambridge, UK) is a validated computerized assessment tool used to measure cognitive function in adults [18].

In this study, it was used to administer the Delayed Match to Sample (DMS), Pattern Recognition Memory (PRM), Reaction Time (RTI), Stockings of Cambridge (SOC), and Spatial Span (SSP) tests. All cognitive tasks were performed in a closed room without any source of external disturbance. The investigator was only present to provide explanation of how to perform the tests. All participants attended familiarization sessions prior to the actual cognitive tests.

The DMS is a test of perceptual matching and immediate and delayed visual memory using a four-choice simultaneous and delayed recognition memory task. The participant is shown a complex visual pattern (i.e., the sample pattern). After a brief delay, the participant is shown four patterns (i.e., choice patterns). In some trials, the sample and the choice patterns are shown simultaneously. In other trials, the sample pattern is shown first, and then 0 seconds, 4 seconds, or 12 seconds later, the choice patterns are shown. A higher percentage of correct responses and shorter latency duration (ms) are indicators of better performance.

The PRM is a test that uses a visual pattern recognition memory in a two-choice forced discrimination task. In this test, a sequence of visual patterns is presented in the center of the screen. In the recognition phase, the participants must choose between a pattern they have already seen and a novel pattern. Lower reaction time and higher accuracy (%) indicate better performance.

The RTI is used to assess attention, based on reaction time. It comprises simple single and multiple-choice reaction time tasks. The participant is instructed to touch the screen when a yellow dot is displayed. For the multiple-choice reaction time task, the dot may appear in one of the five locations. Lower movement time, reaction time, and higher accuracy (%) indicate better performance.

The SOC test is used to assess planning and motor skill ability. The screen is divided into two halves. In the upper half, an image was shown containing three colored balls (red or green or blue). The balls were arranged randomly on three stockings. The CANTAB device displays the minimum number of moves needed to accomplish the task. On the lower display, the participant is required to try to copy the pattern in the top screen by using the touchscreen to move the balls. If the participant completes the problem without exceeding the number of minimum moves, the device records a success and the next problem is displayed. The level of complexity increases. The number of minimum moves for solving the problems randomly varies between two and five moves. The total number of problems the participant solves in the minimum moves out of maximum possible 12 is recorded by the CANTAB device. Generally, a higher number of problems solved in minimum moves and lower mean moves with each complexity indicate better performance.

The SSP is used to determine working memory capacity. The participants are shown 9 white squares arranged on the screen in a misaligned fashion. One at a time, a random square briefly changes color. The participant is required to memorize the sequence and then correctly identify the sequence by using the touchscreen. The sequence and color vary throughout the test, which starts with a span length of one to nine. The longest sequence a person correctly recalls is recorded. The participant can score 0–9, and a maximum score of 9 is indicative of higher memory span length.

### 2.6. Statistical Analysis

All data were coded and analyzed using SPSS (Statistical Package for the Social Sciences) v21.0. The data are presented as mean ± standard deviation (SD) for continuous variables and frequencies for categorical variables. The complex sampling method was applied to randomly selected women in different BMI categories to match them by age. One-way analysis of variance (ANOVA) was performed to determine any association of BMI with performance on the cognitive tasks. The post hoc Bonferroni comparisons were only done whenever a main effect was significant. Spearman's nonparametric correlation coefficient was computed for associations between performance on cognitive tasks with body fat (%) and the measure of cardiorespiratory fitness (VO_2_ max). A *p* value < 0.05 was considered statistically significant.

## 3. Results and Discussion

Out of the 220 participants, 98 participants provided complete data on cognitive assessments. The average age of the participants was 36 ± 8.4 years and ranged from 18 to 50 years. The body fat (%) on average was 46.2 ± 6.6%, and the average determined VO_2_ max was 23.6 ± 6.1 mL/kg/min (Table 1). From the 98 participants, 23.5% were classified as overweight, 22.4% as obese, and 36.7% as morbidly obese, and the rest were found to be included in the normal body weight group.

Table 2 shows the association of BMI categories with various cognitive outcomes like attention, memory, and planning ability. None of the attention parameters (outcomes for movement time and reaction times as well as accuracy for both simple and complex attention tasks) and planning ability tests showed (SOC) any significant difference among the various BMI categories. However, assessment of the association between memory testing parameters and cognitive function among different BMI categories using a one-way ANOVA revealed mixed results.

Although the accuracy on the memory tasks DMS and PRM was higher than 85% and comparable in all BMI categories, the time intervals for correct response expressed as mean correct latency in both DMS (simultaneously and 0 ms delay, *p*=0.005 and 0.045, resp.) and PRM (*p*=0.044) were significantly higher in the morbidly obese groups compared to normal participants. The memory span length on the SSP test was significantly lower among overweight and morbidly obese women compared to women with normal body mass index (*p*=0.011). On the SOC task, the mean number of moves for tests that required only 4 moves was on average 5, and a total number of problems solved in minimum moves were similar across all BMI categories.

It was found in this study that cardiorespiratory fitness was not associated with any of the cognitive outcomes. In addition to BMI, body fat assessed by CT scan and DEXA was tested for the presence of correlations with the cognitive outcomes. Body fat (%) showed similar associations as BMI, and there was a positive correlation of body fat (%) with latency to correct responses (*r* = 0.232 and *r* = 0.229 for DRM and PRM, resp.; *p* < 0.05) but no association with accuracy (Table 3). CT scan images describing the fat accumulation in the regions (heart, liver, abdomen, and thigh) showed no significant association of volume of fat in each region with any of the cognitive parameters (results not shown). Similarly, DEXA-assessed lean body weight and lean mass % did not show any significant difference.

As discussed earlier, the association between obesity (measured by BMI) and cognitive function is not simple and is rather quite complex due to individual, possibly age-related differences. There are evidences from experimental, epidemiological, cross-sectional, and prospective studies that obesity is associated with cognitive decline [18]. Several studies have also confirmed that weight loss or low BMI can lead to decline in cognitive function in elderly population but can be beneficial in younger population in terms of improvement of cognitive function [4, 6–11]. In contrast to the above studies, Elias et al. have found no significant difference in cognitive function in obese and normal body weight female subjects [16].

In our study, the results indicate that obesity was not associated with accuracy on cognitive tasks with regard to attention or planning executive function; however, morbid obesity was significantly associated with a lower memory span and with higher latency to responses. The association of morbid obesity with greater loss of cognitive function is supported by evidences of improvement of cognitive function after bariatric surgery [19]. Moreover, between normal and morbidly obese women, statistical significant differences were found only on latencies that were less than 1 s, suggesting decrements in sensory memory rather than short-term memory. Previous studies demonstrated that obesity was negatively associated with visual acuity (VA) and other eye diseases among adults [20]. Although VA was not measured in the current study, it is possible that these differences observed in sensory memory were associated with decrements in VA in the morbidly obese group. In fact, studies from Henderson et al. [21] and Lindenberger and Ghisletta [22] reported correlations between measures of sensory functions and measures of cognitive functions at different ages.

Gunstad et al. examined attention and executive function among 408 healthy adult persons, aged 20 to 82 years [23]. Similar to our study, this study could not find any significant correlation between attention test performance and BMI. However, executive function test performances showed significant poor performance in overweight and obese that were apparent in the older adults.

Another factor that has been investigated is damage to the hippocampus, which is associated with memory (both short and long terms). Some investigators have found that people with an increased BMI have a reduced hippocampal volume. For example, Cherbuin et al. examined the magnetic resonance images of older individuals (60–64 years), who were followed for an average of 8 years to detect brain changes over time [24]. They assessed the relationship between BMI and hippocampal atrophy and found that BMI was negatively associated with hippocampal volume: people with a higher BMI tended to have greater hippocampal atrophy. A possible reason for this reduction is that excessive amounts of dietary saturated fats may induce changes in the integrity of the blood-brain barrier, which consequently may allow toxins to enter the brain and directly or indirectly impact the hippocampus [25].

In the current study, the volume of brain structures involved in memory was not measured. These factors nevertheless could have impacted our findings. However, DEXA- and CT scan-assessed body fat distribution provided some direct associations of body fat percentage and volume distribution with cognitive function parameters.

Aerobic fitness appears to have a positive effect on cognition [26, 27]. In our study, no association was seen between aerobic fitness and any of the cognitive functions tested. A possible reason for this finding may be related to the fact that the women had been recruited from a gym and were more active, although relatively not aerobically fit. The findings can be linked with practice of regular physical activity that can improve cognition [26]. However, we did not have a sedentary group of women for comparison.

In our study, BMI was negatively associated with some aspects of cognition. We therefore examined the possibility that an increased body fat percentage would similarly show negative impacts on cognition. In the morbidly obese women, body fat was negatively associated with a delayed response on the forced choice recognition memory task (i.e., DMS) and the visual pattern memory task (i.e., PRM). The extent that fat-related factors such as body fat distribution, percentage of body fat, and fat metabolism may be involved in this finding is unclear.

The findings of our study indicate that morbid obesity in otherwise healthy premenopausal women can have a negative impact on recognition memory and memory span length. Future studies are needed on different populations of premenopausal women (e.g., sedentary healthy premenopausal women).

### 3.1. Limitations

There are several limitations of our study: (1) cross-sectional design (assessments were done only once; no follow-up assessments were done to see the pattern of change in cognitive function with BMI over the years). (2) Besides BMI (which is measured in every participant), other parameters of obesity like body fat assessment (measured by CT scan and DEXA scan) were not measured in all the participants (measured only in morbidly obese and obese participants). Perhaps, due to insufficient sample size, CT-assessed body fat volume in various regions (heart, liver, abdomen, and thigh) did not show any significant correlations with cognitive parameters. (3) Visual acuity was not measured in this experiment although participants were allowed to use their glasses which therefore could not have affected our results; future studies investigating the link between obesity and cognitive performance should consider screening for potential decrements in sensory capabilities that may compromise the availability and encoding of relevant information (e.g., sight and hearing).

Our study is among few studies conducted on younger healthy obese and morbidly obese women. Along with BMI, we took into account body fat distribution and aerobic fitness status for any association with cognitive function which is otherwise lacking in literature. All cognitive tasks required finger/hand movements to touch the screen and provide responses. The high latency could be due to the arm movement speed that could be slow in morbidly obese women. The SSP memory span test assesses only short-term memory performance, and our study could not include other domains of memory performance.

## 4. Conclusion

Based on these findings, we conclude that obesity was not associated with cognitive performance related to attention and planning executive function in otherwise healthy premenopausal women; however, morbid obesity is associated with higher latency to correct response on memory-specific tasks and poorer short-term memory. These findings are similar to the findings of studies with similar age groups; however, the absence of link between cardiorespiratory fitness and cognitive function in this study suggests other pathways responsible for cognitive decline.

## Figures and Tables

**Table 1:** Characteristics of subjects.

	Number of subjects	Mean	Standard deviation	Median	Minimum	Maximum
Age (years)	98	36.00	8.44	37.00	18.00	50.00
Height (cm)	98	158.67	5.19	158.35	146.00	169.60
Weight (kg)	98	81.84	19.32	80.45	47.00	127.40
Body mass index (kg/m^2^)	98	32.6	7.69	31.12	17.84	49.30
Waist circumference (cm)	91	96.68	14.67	94.00	64.00	135.00
VO_2_ max (Bruce test)	74	23.67	6.12	24.46	9.24	41.07
Body fat (%)	96	46.16	6.56	46.80	29.00	56.90

**Table 2:** Association of BMI categories with cognitive outcomes (attention, memory, and planning ability).

Name of CANTAB test and cognitive outcomes	Body mass index category (kg/M^2^)				*p* value^∗^
	Normal (*n*=17)	Overweight (*n*=23)	Obese (*n*=22)	Morbidly obese (*n*=36)	
*Reaction Time (RTI) for attention*					
Five-choice movement time (ms)	334.7 ± 103.8	371.5 ± 84.8	332.9 ± 70.6	338.8 ± 111.6	0.756
Five-choice reaction time (ms)	320.6 ± 43.6	342.1 ± 31.5	331.1 ± 42	351.4 ± 54.1	0.253
Five-choice accuracy (%)	98.8 ± 2.7	100 ± 0	100 ± 0	97.5 ± 5.4	0.175
Simple movement time (ms)	355.5 ± 134.9	408.4 ± 141.1	392.9 ± 172.3	340.6 ± 129	0.568
Simple reaction time (ms)	307.8 ± 76.7	305.5 ± 24.1	322.9 ± 86.9	323.6 ± 63.8	0.645
*Delayed Match to Sample (DMS) for memory*					
Mean correct latency (ms)	3163.1 ± 653.8	3321.5 ± 782.1	3473.5 ± 826.8	3715.4 ± 915.3	0.117
Mean correct latency (all delays) (ms)	3326.3 ± 627.7	3429.8 ± 822.9	3556.7 ± 853.7	3792.6 ± 1091.5	0.342
Mean correct latency (simultaneous) (ms)	2713.1 ± 939.8	3051.6 ± 876.5	3204.7 ± 1410.1	3498.2 ± 871.8^∗^	0.005
Mean correct latency (0 ms delay) (ms)	2724.9 ± 767.4	2776.3 ± 650.1	2835.9 ± 561.4	3312.6 ± 1165.6^∗^	0.045
Mean correct latency (4000 ms delay) (ms)	3219.7 ± 1013.5	3886 ± 1275.3	3518.9 ± 1068.4	3928.2 ± 1411	0.165
Mean correct latency (12000 ms delay) (ms)	4149.4 ± 644.9	3585.2 ± 953.9	4197.7 ± 1884.8	4092.6 ± 1460.2	0.228
Percent correct (%)	88.6 ± 12.1	85.4 ± 9	85.9 ± 9.5	86.6 ± 10.3	0.445
Percent correct (all delays) (%)	85.1 ± 15.6	81.7 ± 12.1	81.5 ± 12.3	82.6 ± 13.4	0.511
Percent correct (simultaneous) (%)	98.8 ± 4.9	96.5 ± 9.8	99.1 ± 4.3	98.3 ± 5.6	0.728
Percent correct (0 ms delay) (%)	85.6 ± 18.5	85.2 ± 16.2	79.8 ± 13.8	83.9 ± 19	0.436
Percent correct (4000 ms delay) (%)	87.1 ± 21.1	87 ± 14.3	89.4 ± 12.8	85.4 ± 14.9	0.679
Percent correct (12000 ms delay) (%)	83.5 ± 19	73 ± 23	75.5 ± 25.4	78.5 ± 24.4	0.476
*Pattern Recognition Memory (PRM)*					
Mean correct latency (ms)	2301.9 ± 824.2	2967.4 ± 1667.5	2629.9 ± 1034.6	2868.1 ± 995.5^∗^	0.044
Percent correct (%)	92.2 ± 6.4	85.7 ± 12.7	88.6 ± 10.1	85 ± 14	0.182
*Spatial Span (SSP) for memory*					
Span length (1–9)	6.7 ± 0.9	5.5 ± 1.3^∗^	6.1 ± 1.5	5.6 ± 1.6^∗^	0.011
Total errors	17.6 ± 4.7	13.5 ± 5	14.6 ± 7.6	14.3 ± 6.5	0.082
Total usage errors	2.4 ± 1.9	2.4 ± 1.6	1.9 ± 1.3	2.7 ± 2.2	0.670
*Stockings of Cambridge (SOC) for planning ability*					
SOC mean moves (2 moves)	2.1 ± 0.2	2.2 ± 0.5	2.1 ± 0.2	2.2 ± 0.4	0.485
SOC mean moves (3 moves)	3.5 ± 0.9	3.7 ± 0.9	3.6 ± 0.7	3.6 ± 1	0.618
SOC mean moves (4 moves)	5 ± 1.1	5 ± 1	5.3 ± 1.4	5 ± 1.1	0.784
SOC problems solved in minimum moves (0–12)	5 ± 1.2	5 ± 1.5	4.7 ± 1.1	5.2 ± 1.5	0.549

ms: milliseconds; accuracy/correct (%) ranges from 0 to 100%. Span length is a discrete value (ranging from 1 to 9): the longest sequence participant correctly recalled. SOC parameters are all discrete. Mean move for 2, 3, or 4 is the average number of moves a participant takes to solve a problem that requires only 2, 3, or 4 moves, respectively. SOC: problems solved in minimum moves out of maximum possible 12. ^∗^Significantly different from Normal BMI group (*p* ≤ 0.05).

**Table 3:** Correlation of cognitive parameters assessed using CANTAB with body mass index, body fat (%), and VO_2_ max.

Name of CANTAB test and cognitive outcomes	BMI (kg/m^2^) *r*	VO_2_ max *r*	Body fat (%) *r*
*Reaction Time (RTI) for attention*			
Five-choice accuracy score (%)	−0.04	0.06	−0.05
Five-choice movement time (ms)	−0.03	−0.08	−0.03
Five-choice reaction time (ms)	0.25	−0.28	0.27
Simple movement time (ms)	−0.03	−0.08	−0.14
Simple reaction time (ms)	0.18	−0.14	0.18
*Delayed Match to Sample (DMS) for memory*			
Mean correct latency (ms)	0.248^∗^	−0.11	0.18
Mean correct latency (all delays) (ms)	0.17	−0.06	0.15
Mean correct latency (simultaneous) (ms)	0.377^∗∗^	−0.17	0.232^∗^
Mean correct latency (0 ms delay) (ms)	0.214^∗^	0.02	0.15
Mean correct latency (4000 ms delay) (ms)	0.13	0.01	0.09
Mean correct latency (12000 ms delay) (ms)	0.01	−0.13	0.05
Percent correct (%)	−0.05	0.02	0.03
Percent correct (all delays) (%)	−0.06	0.05	0.02
Percent correct (simultaneous) (%)	0.03	−0.12	0.01
Percent correct (0 ms delay) (%)	−0.02	0.08	0.01
Percent correct (4000 ms delay) (%)	−0.13	0.13	−0.08
Percent correct (12000 ms delay) (%)	0.03	−0.09	0.09
*Spatial Span (SSP) for memory*			
Span length (1–9)	−0.16	−0.05	−0.08
Total errors	−0.14	0.02	−0.13
Total usage errors	0.06	0.15	0.04
*Pattern Recognition Memory (PRM)*			
Mean correct latency (ms)	0.209^∗^	−0.16	0.229^∗^
Percent correct (%)	−0.08	−0.02	−0.12
*Stockings of Cambridge (SOC) for planning ability*			
Mean moves (2 moves)	0.04	−0.23	0.04
Mean moves (3 moves)	−0.05	0.07	−0.06
Mean moves (4 moves)	0.06	0.05	−0.01
Problems solved in minimum moves (0–12)	0.06	0.05	0.04

*r*: correlation coefficient; ^∗∗^correlation is significant at the 0.01 level (2-tailed). ^∗^Correlation is significant at the 0.05 level (2-tailed). ms: milliseconds; accuracy/correct (%) ranges from 0 to 100%. Span length is a discrete value (ranging from 1 to 9): the longest sequence participant correctly recalled. SOC parameters are all discrete. Mean move for 2, 3, or 4 is the average number of moves a participant takes to solve a problem that requires only 2, 3, or 4 moves, respectively. SOC: problems solved in minimum moves out of maximum possible 12.
